# Effects of early postpartum massage on physical discomfort, mood, and emotional well-being: A randomized controlled trial

**DOI:** 10.18332/ejm/216378

**Published:** 2026-01-31

**Authors:** Ai Yamasaki, Nobuko Sakamoto, Yuka Edamitsu, Mitsuko Ishibashi, Fuminori Kimura, Toshiko Igarashi

**Affiliations:** 1 Department of Women’s Health and Midwifery, Graduate School of Nursing, Nara Medical University, Nara, Japan; 2 Department of Maternity Nursing and Midwifery, Nara Medical University Hospital, Nara, Japan; 3 Department of Obstetrics and Gynecology, Nara Medical University, Nara, Japan

**Keywords:** early postpartum, massage, unpleasant symptoms, moods, emotions, POMS^®^2

## Abstract

**INTRODUCTION:**

Postpartum women commonly experience interrelated unpleasant physical symptoms and negative emotions, necessitating a comprehensive approach that addresses both physiological and psychological well-being. In this study, we aimed to assess the effectiveness of massage therapy in alleviating unpleasant symptoms and improving mood and emotional states in the early postpartum period.

**METHODS:**

A randomized controlled trial was conducted in 2024 at a single-center facility involving 112 women aged ≥18 years who had undergone vaginal delivery, could read and write Japanese, and had no musculoskeletal or psychiatric disorders. Participants were randomly assigned to either an intervention group, which received massage therapy on the second postpartum day, or a control group undergoing bed rest. Both groups completed pre- and post-intervention assessments on physical symptoms and mood/emotions, while demographic and clinical data were obtained from medical records.

**RESULTS:**

A comparative analysis of using t-tests pre- and post-intervention changes between the two groups revealed statistically significant differences in four physical symptoms, five negative mood/emotion subscales, and two positive mood/emotion subscales (p<0.05). Multiple regression analysis was adopted, adjusting for six key variables: intervention status, primiparity, duration of labor, blood loss during delivery, weeks postpartum, and mother–infant separation. The results identified massage therapy as the factor for improving early postpartum discomfort and emotional well-being (p<0.05).

**CONCLUSIONS:**

This study demonstrated that massage therapy in the early postpartum period is effective in alleviating unpleasant symptoms. Additionally, massage was found to reduce negative mood states, while simultaneously enhancing positive emotions.

**CLINICAL TRIAL REGISTRATION:**

The study is registered on the official website of UMIN Clinical Trials Registry.

**IDENTIFIER:**

UMIN000052795

## INTRODUCTION

The postpartum period is a critical phase for maternal recovery^1^ during which the body heals and regains strength following pregnancy and childbirth. Several factors contribute to a decline in health-related quality of life (QOL) during this period, including physical issues such as pain and discomfort, as well as psychological factors such as anxiety and depression^2^. Particularly in the early postpartum phase, women frequently experience health concerns such as fatigue, backache, bowel problems, interrupted sleep, hemorrhoids, and perineal pain^3^. Additionally, this period is characterized by postpartum-specific psychological states, including depressive moods and anxiety, with maternity ‘blues’ being a representative condition^4^.

A Japanese study involving postpartum women demonstrated a significant increase in the prevalence of physical symptoms between the immediate postpartum period and one month after childbirth (p<0.001), including shoulder stiffness (50.4% to 66.6%), fatigue (47.2% to 54.3%), difficulty in returning to pre-pregnancy body shape (46.2% to 51.8%), eye strain (20.2% to 28.4%), tenosynovitis (5.6% to 27.5%), and headache (10.0% to 24.8%)^5^. Notably, shoulder stiffness is highly prevalent, with a reported incidence of 73.1% at one month postpartum^6^; however, few studies have investigated shoulder stiffness in the early postpartum period^7^.

Previous research has shown that 38.8% of women experience fatigue or severe fatigue by postpartum day ten^8^. Postpartum fatigue is linked to childcare-related stress, sleep disturbances, bodily discomfort, and emotional dysregulation, all of which are known risk factors for postpartum depression^9^. Moreover, depressive symptoms within 24 hours postpartum have been found to correlate with depression at four months postpartum^10^. Women who experience maternity ‘blues’ are also considered to be at an increased risk of developing postpartum depression^11^.

Physical and psychological health issues in the early postpartum period not only affect maternal QOL but also contribute to the development of postpartum depression^2^. Thus, it is essential to address both physical discomfort and psychological distress as early as possible.

Among complementary health approaches for managing postpartum pain, acupuncture, massage, herbal ointments, cupping (including shiatsu) and herbal tablets have been identified as effective interventions^12^. In particular, back massage has been demonstrated to alleviate low back pain^13^, while exercise and herbal tea consumption have been reported to reduce postpartum fatigue^14,15^.

It has been hypothesized that the administration of back massage during the postpartum period may reduce anxiety levels in primiparous women^16^ and induce psychological relaxation^17^. Furthermore, back massage is believed to be effective in addressing mental health concerns among postpartum women. However, the majority of existing interventions are implemented within the first week of the postpartum period, with durations ranging from several days to several weeks. To our knowledge, no studies have specifically investigated the effects of a single-session intervention during the early postpartum period.

Persistent or worsening discomfort symptoms in the early postpartum period may exacerbate negative emotions^18^, while heightened negative emotions can, in turn, increase the perception of physical discomfort^19^, demonstrating a bidirectional relationship between these factors. Given this interdependence, addressing both physical and psychological issues is important in the early postpartum period.

The present study aimed to examine the effectiveness of an early postpartum intervention in alleviating physical and psychological burdens by investigating whether focused massage on the upper body during the early postpartum period (postpartum day 2) reduces unpleasant physical symptoms, such as shoulder stiffness, as well as negative moods and emotions, including depression.

## METHODS

### Study design and sample

This study is a single-center randomized controlled trial comparing two groups: an intervention group receiving massage therapy through random allocation and a control group undergoing bed rest.

The study was conducted at a university hospital in Nara Prefecture, Japan, which serves as a comprehensive perinatal, maternal, and child medical center. The massage therapy, provided by Eclore Co., Ltd., is administered to postpartum women hospitalized during the postpartum period.

The study population consists of postpartum women admitted to the research facility for postpartum care between January and July 2024, on the second day following vaginal delivery. Individuals were excluded for the following reasons: aged <18 years; insufficient Japanese language proficiency to comprehend the study protocol; documented mental health conditions; musculoskeletal disorders; and those deemed unsuitable for study participation due to other medical complications as assessed by healthcare professionals.

In a previous study examining the effects of back massage in postpartum women, the mean POMS^®^ score was reported as 183.3 (SD=41.0) before the intervention and 168.2 (SD=33.1) after the intervention^20^. Based on these data, the required sample size was calculated to be 45 participants, assuming a two-sided significance level of 5% (α=0.05) and a statistical power of 80% (1-β = 0.80). Allowing for an anticipated dropout rate of 10%, the final target sample size was set at 100 participants, with 50 participants allocated to each group.

### Instruments

*Patient information form*

Demographic and clinical information, including age, weeks postpartum, duration of delivery, blood loss during delivery, parity, and whether mother–infant separation occurred, was collected from medical records.

*Assessment of unpleasant symptoms*

Unpleasant symptoms commonly experienced during the postpartum period were selected based on previous literature^5^ and postpartum women’s feedback on massages conducted at the research facility. The identified symptoms included shoulder stiffness, fatigue/lethargy, eye fatigue, arm fatigue, and headache, all of which were measured using a five-point Likert scale ranging from ‘not at all’ to ‘very much’.

*Assessment of mood and emotions*

Mood and emotional states were assessed using the Japanese version of the Profile of Mood States, Second Edition (POMS^®^2). The POMS^®^2 is a validated questionnaire-based mood profile test that evaluates recent mood and emotional states using a five-point scale (not at all, a little, a fair amount, quite a lot) across seven subscales: anger-hostility (AH), confusion-bewilderment (CB), depression-dejection (DD), fatigue-inertia (FI), tension-anxiety (TA), vigor-activity (VA), and friendliness (F)^21^.

This study employed the short-form version of the POMS^®^2, which consists of 35 items and can be completed in approximately five minutes. Raw scores from the seven subscales were converted to standardized T-scores derived from a large, representative normative sample. The Japanese version of the short-form POMS^®^2 has demonstrated high reliability, with a Cronbach’s alpha coefficient ranging from 0.79 to 0.96, and its validity has been confirmed^21^.

### Randomization

Random allocation of participants to the intervention and control groups was performed by a blinded study investigator. Given the potential impact of delivery duration^22^ and the effects of childcare experience^23^, separate random number tables were generated for primiparous and multiparous women. Participants were then assigned to the intervention and control groups using a permuted block randomization method. This was an open-label randomized controlled trial. Due to the nature of the care-based intervention, blinding of participants, intervention providers, and outcome assessors was not feasible.

### Data collection

Following vaginal delivery, eligible participants who provided written and oral informed consent were randomly assigned to either the intervention or control group. The pre- and post-surveys were conducted on the second postpartum day, with childcare support provided during the assessment period. Eligible participants were hospitalized in private rooms. Both groups completed electronic surveys via tablets before and after the intervention.

*Intervention group*

Participants in the intervention group received a 40-minute massage administered by a trained practitioner from Eclore Co., Ltd. The massage technique involves the application of massage oil and the use of the therapist’s palms and fingers to perform effleurage and kneading on the upper body (from the collarbone to the shoulders and upper limbs), back (below the shoulder blades), lower limbs, and head. To ensure consistency and standardization, the massage is exclusively administered by therapists who have completed a rigorous training program and received standardized technical education.

*Control group*

Participants in the control group rested in a supine position for 40 minutes. From the third postpartum day onward, they received the same massage treatment as the intervention group.

### Ethical considerations

Participants were informed of the ethical considerations both verbally and in writing. Personal data were anonymized to ensure confidentiality. The study protocol was approved by the Medical Ethics Committee of Nara Medical University (approval number: 3699). The trial was registered with the UMIN Clinical Trials Registry (UMIN000052795).

### Data analysis

The analysis was conducted according to an intention-to-treat. Descriptive statistics, including means, standard deviations, frequencies, and percentages, were used to summarize the data. Formal tests of normality were not conducted due to the adequate sample size (n=112); parametric analyses were applied based on the central limit theorem and supported by graphical inspection of data distributions.

To assess changes before and after the intervention, paired t-tests were conducted to compare mean values between the intervention and control groups for the five unpleasant symptoms commonly experienced during the postpartum period and the seven POMS^®^2 subscales.

To identify factors influencing the alleviation of unpleasant symptoms and mood disturbances, a multiple regression analysis was performed using four dependent variables: shoulder stiffness; fatigue/lethargy; POMS^®^2 depression/dejection (DD) score, and the POMS^®^2 fatigue/inertia (FI) score. The independent variables included: primiparity, delivery duration ≥8 hours (the cutoff value was the median delivery time for Japanese primiparas^24^); blood loss at delivery ≥500 g (definition of atonic bleeding in Japan^25^), and the presence or absence of mother–infant separation. Statistical analyses were conducted using IBM SPSS Statistics version 28.0 (IBM Corp., Armonk, NY, USA) with a significance threshold set at p<0.05. Due to the nature of the intervention, blinding of participants, intervention providers, and analysts to group allocation was not feasible.

## RESULTS

### Sample characteristics

**Figure 1:**
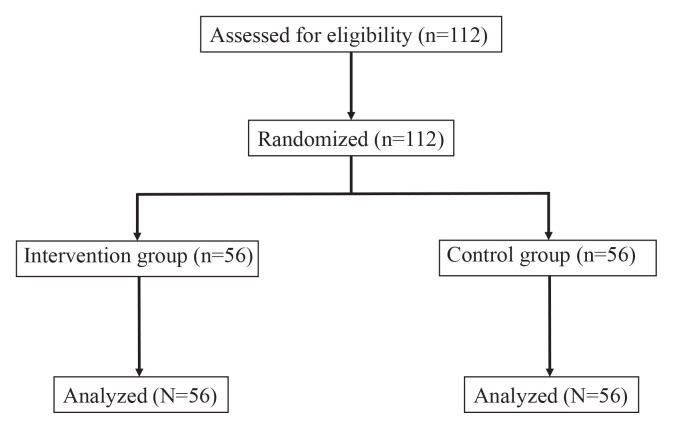
Participant flowchart

Of the 113 participants who provided informed consent to participate in the study, one was excluded due to missing data, resulting in a final sample of 112 participants: 56 in the intervention group and 56 in the control group (Figure 1 and Table 1).

**Table 1:** Baseline characteristics of participants on postpartum day 2 at Nara Medical University Hospital in Japan, January–July 2024 (N=112)

*Characteristics*	*Intervention group* *(N=56)*	*Control group* *(N=56)*	
	*Mean (SD)*	*Mean (SD)*	*p^†^*
Age (years)	32.2 (4.4)	32.6 (5.3)	0.66
Gestational age (days)	271.7 (11.7)	271.8 (10.9)	0.96
Delivery time (min)	480.5 (377.8)	482.2 (408.4)	0.98
Blood loss during delivery (g)	551.8 (380.4)	604.9 (387.3)	0.47
	***n%***	***n%***	***p^‡^***
Age at birth: <37 weeks	6 (10.7)	6 (10.5)	1.00
Delivery time: ≥8 hours	18 (32.1)	17 (30.4)	0.83
Blood loss during delivery: ≥500 g	23 (41.1)	30 (54.3)	0.19
Primipara	28 (50.0)	28 (50.0)	1.00
Separation of mother and child possible	10 (17.9)	7 (12.3)	0.43

† t-test.

‡ χ² test.

### Comparison of changes in unpleasant symptoms between groups

In both groups, the mean scores for all six categories of unpleasant symptoms decreased from pre- to post-intervention. Prior to the intervention, the highest-rated unpleasant symptom in both groups was ‘fatigue and lethargy’, followed by ‘leg swelling’.

In the intervention group, the symptom that exhibited the greatest mean change (SD) from pre- to post-intervention was ‘fatigue and lethargy’ (-1.82 ± 1.25), followed by ‘leg swelling’ (-1.37 ± 1.24). Similarly, in the control group, the most substantial mean change (SD) was observed for ‘fatigue and lethargy’ (-0.50 ± 0.93), followed by ‘eye fatigue’ (-0.41 ± 0.95). For all symptoms, the mean change in scores was greater in the intervention group than in the control group.

A comparison of pre- and post-intervention mean changes in common postpartum symptoms revealed statistically significant differences between the two groups for five of the six symptoms, with ‘stiff shoulders’ (p<0.00), ‘fatigue-dullness’ (p<0.00), ‘arm fatigue’(p<0.00), ‘eye fatigue’ (p<0.00), ‘swelling of the legs’ (p<0.00) (Table 2), demonstrating that the intervention was effective in reducing postpartum discomfort.

**Table 2:** Comparison of changes in unpleasant symptoms mean scores in both groups at baseline and after the intervention (N=112)

*Variables*	*Intervention group (N=56)*			*Control group (N=56)*			Changes p*
	*Before*	*After*	*Changes* *Mean (SD)*	*Before*	*After*	*Changes* *Mean (SD)*	
Stiff shoulders	1.93	0.77	-1.16 (1.26)	1.71	1.36	-0.36 (0.75)	<0.001
Fatigue and dullness	2.41	0.59	-1.82 (1.25)	2.11	1.61	-0.50 (0.93)	<0.001
Arm fatigue	1.45	0.45	-1.00 (1.00)	1.43	1.13	-0.30 (0.87)	<0.001
Eye fatigue	1.66	0.48	-1.18 (1.11)	1.36	0.95	-0.41 (0.95)	<0.001
Headache	0.43	0.05	-0.37 (0.82)	0.34	0.20	-0.14 (0.52)	0.07
Swelling of the legs	2.16	0.79	-1.37 (1.24)	1.91	1.52	-0.39 (0.76)	<0.001

* Significant at p<0.05; t-tests.

### Comparison of changes in POMS®2 mean scores between groups

In both groups, the baseline T-scores for the seven POMS^®^2 subscales were within the normal range, as per standardized guidelines^21^.

In the intervention group, mean scores decreased from pre- to post-intervention for five negative emotion subscales: ‘anger-hostility (AH)’, ‘confusion-bewilderment (CB)’, ‘depression-dejection (DD)’, ‘fatigue-inertia (FI)’, and ‘tension-anxiety (TA)’. Conversely, mean scores increased for two positive emotion subscales: ‘vigor-activity (VA)’ and ‘friendliness (F)’. The largest mean change (SD) was observed for ‘tension-anxiety’ (-12.38 ± 7.60), followed by ‘fatigue-inertia’ (-9.11 ± 8.73).

In the control group, mean scores also decreased from pre- to post-intervention across all seven POMS^®^2 subscales. The subscales with the greatest mean change (SD) were the same as those in the intervention group: ‘tension-anxiety (TA)’ (-7.07 ± 5.95), followed by ‘fatigue-inertia (FI)’ (-5.18 ± 6.63).

A comparison between the intervention and control groups indicated that the reduction in mean scores for the five negative emotion subscales was greater in the intervention group. Additionally, the difference in mean score changes between the two groups was statistically significant for all seven POMS^®^2 subscales, with ‘anger-hostility (AH)’ (p<0.02), ‘confusion-bewilderment (CB)’ (p<0.00), ‘depression-dejection (DD)’ (p<0.00), ‘fatigue-inertia (FI)’ (p<0.00), ‘tension-anxiety (TA)’ (p<0.00), ‘vigor-activity (VA)’ (p<0.00), ‘friendliness(F)’ (p<0.00) (Table 3).

**Table 3:** Comparison of changes in POMS^®^2 mean scores of both groups at baseline and after the intervention (N=112)

*Variables*	*Intervention group (N=56)*			*Control group (N=56)*			Changes p*
	*Before*	*After*	*Changes* *Mean (SD)*	*Before*	*After*	*Changes* *Mean (SD)*	
Anger-Hostility (AH)	44.0	38.9	-5.13 (7.33)	42.6	40.3	-2.25 (5.36)	0.02
Confusion-Bewilderment (CB)	50.3	42.7	-7.63 (7.94)	47.7	44.8	-2.91 (5.45)	0.00
Depression-Dejection (DD)	47.9	43.4	-4.54 (5.86)	46.9	45.0	-1.91 (3.15)	0.00
Fatigue-Inertia (FI)	47.5	38.4	-9.11 (8.73)	48.6	43.4	-5.18 (6.63)	0.00
Tension-Anxiety (TA)	53.1	40.8	-12.38 (7.60)	51.4	44.3	-7.07 (5.95)	0.00
Vigor-Activity (VA)	50.0	55.9	5.80 (7.61)	49.3	47.3	-2.07 (5.48)	0.00
Friendliness (F)	49.8	51.7	2.54 (9.28)	49.4	46.2	-3.12 (4.98)	0.00

* Significant at p<0.05; t-tests.

### Factors influencing unpleasant symptoms and mood/emotions

To identify factors influencing changes in four key variables ‘shoulder stiffness’, ‘fatigue-lethargy’, ‘depression-dejection (DD)’ , and ‘fatigue-inertia (FI)’ on POMS^®^2, a multiple regression analysis was performed. The independent variables included: intervention status (whether or not the massage intervention was received), primiparity, delivery duration of ≥8 hours, blood loss at delivery of ≥500 g, and mother–infant separation. The analysis revealed that ‘whether or not the intervention was performed’ was the only factor that significantly influenced changes in all four outcome variables (Supplementary file Tables 1–4).

## DISCUSSION

The findings of this study indicate that both the intervention group, which received massage therapy on the second postpartum day, and the control group, which remained on bed rest, exhibited improvements in six unpleasant symptoms and five out of the seven POMS^®^2 subscales measuring negative mood and emotions. Furthermore, a significant difference was observed between the two groups in the magnitude of change before and after the intervention.

Additionally, even after adjusting for potential confounding factors – including whether or not the participant received a massage, primiparity, delivery duration of ≥8 hours, blood loss at delivery of ≥500 g, and mother–infant separation – the factor that had a statistically significant impact on the improvement of ‘stiff shoulders’, ‘fatigue-dullness’, ‘depression-dejection (DD)’, and ‘fatigue-inertia (FI)’ was the presence or absence of the massage intervention.

### The effect of massage on unpleasant symptoms

In both the intervention and control groups, a reduction in discomfort symptoms was observed from pre- to postsurvey. This suggests that even 40 minutes of bed rest can contribute to symptom relief in the early post-partum period. However, a comparison between the intervention and control groups revealed a statistically significant difference in the magnitude of improvement for five symptoms, with the exception of ‘headache’, indicating that massage therapy was more effective than bed rest in alleviating unpleasant symptoms.

Among the six unpleasant symptoms assessed, ‘fatigue-dullness’ and ‘leg swelling’ had the highest baseline scores in both groups, and they also exhibited the largest changes from pre- to post-survey. A previous study^5^ reported that the most frequently experienced postpartum symptoms during hospitalization were ‘stiff shoulders’, followed by ‘fatigue-dullness’ and ‘swelling’. While staff at the research facility noted that many postpartum women complained of ‘stiff shoulders’, the findings of this study differed from previous reports.

Prior research has indicated that postpartum ‘stiff shoulders’ typically develop around 8.1 ± 5.9 days postpartum, which is later than the timing at which participants in this study were assessed. Furthermore, previous studies have demonstrated that pain and unfamiliar childcare practices increase fatigue among postpartum women within 48 hours after childbirth^26^. In this study, outcomes were assessed over a short period, such as the second day after delivery, and the symptoms of discomfort were subjective in nature, so it is possible that symptoms that are easier for postpartum women to recognize were ranked higher than localized, isolated discomfort symptoms such as ‘stiff shoulders’.

Previous research has shown that stretching and strengthening exercises during pregnancy can alleviate ‘stiff shoulders’ in the prenatal period^27^, and that massage therapy is effective in relieving muscle tension in the shoulder region for both men and women^28^. The significant improvement observed in the intervention group in this study is likely attributed to the enhanced blood circulation in the neck and shoulders and muscle relaxation experienced by postpartum women following massage therapy.

In this study, it was hypothesized that the high baseline scores for ‘fatigue-dullness’ could be attributed to the implementation of rooming-in practices from the day of birth at the research facility. Postpartum women are known to experience increased fatigue and sleep disturbances, with reduced nighttime sleep and increased daytime sleep, aligning with the newborn’s sleep–wake cycle^29^. It is likely that the high pre-survey scores for ‘fatigue-dullness’ resulted from a combination of postpartum recovery from childbirth-related exhaustion and sleep deprivation due to adjusting to nighttime infant care.

Several interventions have been identified as effective in alleviating postpartum fatigue, including herbal tea consumption, warm showers, and aromatherapy baths with lavender oil^13^. However, findings from this study suggest that massage therapy had a muscle-relaxing effect similar to the fatigue-reducing benefits of progressive muscle relaxation techniques^27,28^, leading to improvements in ‘fatigue-dullness’. Moreover, the massage technique utilized in this study could be performed while the patient was in a supine position, making it a safe and feasible intervention for women in the early postpartum period.

### Effects of massage on mood and emotions

The findings of this study indicate that massage therapy was effective in alleviating negative emotions and enhancing positive emotions in the early postpartum period. Notably, the two subscales that exhibited the greatest changes – ‘tension-anxiety (TA)’ and ‘fatigue-inertia (FI)’ – are consistent with prior research demonstrating that slow back massage can alleviate these symptoms^16,30^. Additionally, a study utilizing the POMS^®^ scale, similar to the present study, reported that whole-body aromatherapy massage administered on the second postpartum day significantly improved mood and emotions^31^.

Regarding the psychological effects of massage therapy in the context of sports and athletic performance, previous studies have found a positive correlation between massage and improved psychological state, enhanced mood, relaxation, and fatigue recovery^32^. Similarly, the massage intervention in this study was effective in promoting relaxation and fatigue recovery and in reducing negative mood among postpartum women.

On the other hand, while the change in ‘depression-dejection (DD)’ was statistically significant in the intervention group, it exhibited the smallest change among the five negative mood subscales in both groups. This may be explained by previous findings indicating that maternity ‘blues’, characterized primarily by symptoms of depression and low mood, peak around the fifth postpartum day^33^. Consequently, a few postpartum women on the second day after childbirth may have been consciously aware of feelings of depression. However, depression and anxiety have been reported to be correlated within the first 24 hours postpartum^10^. Given that ‘tension-anxiety (TA)’ had the highest baseline score in this study, it can be inferred that while participants may not have explicitly identified feelings of depression, they were potentially at risk for developing depressive symptoms.

Moreover, early postpartum depression has been linked to depressive symptoms persisting for several months postpartum^4,10^, highlighting the importance of early interventions to mitigate negative mood and emotions. The massage intervention in this study may therefore play a preventive role in reducing the risk of postpartum depression.

This study also found that positive mood and emotions, such as ‘vigor-activity (VA)’ and ‘friendliness(F)’, increased from pre- to post-massage in the intervention group but not in the control group. This effect may be attributed to the interaction with the massage therapist, including the warmth of human touch and the unique sensory experience of massage, which cannot be achieved through rest alone and may have contributed to increased relaxation. Previous research has shown that positive emotions enhance ego resilience and improve overall life satisfaction^34^. Given that the early postpartum period involves profound physical and emotional transitions – such as recovery from childbirth and the initiation of childcare – promoting positive emotions through massage therapy may be particularly beneficial.

### Effects of massage in the early postpartum period

Primiparous women are more likely than multiparous women to experience fatigue due to their unfamiliarity with childcare^35^, while factors such as a prolonged second stage of labor and perineal pain have been shown to exacerbate postpartum fatigue^36^. Additionally, mother–infant separation due to preterm birth or neonatal intensive care unit (NICU) admission has been associated with increased maternal depressive symptoms, with maternal anxiety levels rising as gestational age decreases^37^.

Despite these varying risk factors, this study demonstrated significant improvements in unpleasant symptoms and in mood and emotions, even after adjusting for parity, duration of labor, and mother–infant separation. Given that postpartum hospitalization is generally brief, typically lasting approximately five days, the findings of this study indicate that massage therapy provided during hospitalization may contribute to reductions in physical discomfort and negative emotional states, regardless of individual maternal risk factors, suggesting its potential role in immediate postpartum care.

### Limitations

This study assessed participants on the second postpartum day; however, given that shoulder stiffness and depressive symptoms tend to peak later, it may be necessary to reconsider the optimal timing for massage interventions. Future research should explore whether delaying the intervention slightly may yield even greater benefits. Additionally, this study was a one-off study, with a very short time between intervention and outcome measurement, so it is unclear whether the effects will last. Furthermore, the present study was limited to evaluating the impact of selected risk factors on the participants and did not assess other unpleasant symptoms, such as sleep and eating disturbances, nor their associated psychological effects. While, previous research has indicated that multiple sessions over several months are effective as non-pharmacological interventions for reducing postpartum fatigue and anxiety^15,38,39^. Future studies should investigate the optimal duration and frequency of massage interventions to maximize their therapeutic efficacy. Due to the nature of the intervention, blinding of participants was not feasible, which may have increased the risk of bias.

## CONCLUSIONS

This study demonstrated that massage therapy in the early postpartum period is effective in alleviating unpleasant symptoms, including ‘stiff shoulders’, ‘fatigue-lethargy’, ‘eye fatigue’, and ‘arm fatigue’. Additionally, massage was found to reduce negative mood states, such as ‘anger-hostility (AH)’, ‘confusion-bewilderment (CB)’, ‘depression-dejection (DD)’, ‘fatigue-inertia (FI)’, and ‘tension-anxiety (TA)’, while simultaneously enhancing positive emotions, including ‘vigor-activity (VA)’ and ‘friendliness (F)’. This study assessed an early, short-term intervention implemented during postpartum hospitalization. Further research is needed to clarify appropriate implementation methods and intervention duration to ensure long-term effectiveness.

## Supplementary Material



## Data Availability

The data supporting this research cannot be made available for privacy or other reasons.
