# Supplementary material for: Effects of early postpartum massage on physical discomfort, mood, and emotional well-being: A randomized controlled trial
Source: Eur J Midwifery. 2026 Jan 31;10:10.18332/ejm/216378. doi: 10.18332/ejm/216378 (PMC12859971; doi:10.18332/ejm/216378)
Supplement: Supplementary file 1 [file EJM-10-03-s1.pdf]

**Supplementary Table 1. Factors that affect the amount of change at baseline and after the intervention〈Stiff shoulders〉**

| Explanatory variable | Partial regression coefficient | Standard partial regression coefficient | P-value | 95%CI | |
|---|---|---|---|---|---|
| | | | | Min | Max |
| constant | -0.24 | | 0.36 | -0.76 | 0.28 |
| Intervention or not | -0.80 | -0.36 | 0.00* | -1.19 | -0.40 |
| Primipara | 0.01 | 0.01 | 0.96 | -0.43 | 0.46 |
| Delivery time: ≧8 hours | -0.15 | -0.06 | 0.53 | -0.61 | 0.32 |
| Blood loss during delivery: ≧500g | -0.19 | -0.04 | 0.69 | -0.51 | 0.34 |
| Separation of mother and child possible | -0.27 | -0.96 | 0.34 | -0.83 | 0.29 |

$R^2 = 0.38$ ANOVA $*p < 0.05$

**Supplementary Table 2. Factors that affect the amount of change〈Fatigue and dullness〉**

| Explanatory variable | Partial regression coefficient | Standard partial regression coefficient | P-value | 95%CI | |
|---|---|---|---|---|---|
| | | | | Min | Max |
| constant | -0.43 | | 0.13 | -0.98 | 0.12 |
| Intervention or not | -1.29 | -0.51 | 0.00* | -1.72 | -0.87 |
| Primipara | -0.17 | -0.07 | 0.48 | -0.64 | 0.30 |
| Delivery time: ≧8 hours | -0.03 | -0.01 | 0.90 | -0.52 | 0.46 |
| Blood loss during delivery: ≧500g | 0.10 | 0.04 | 0.65 | -0.34 | 0.55 |
| Separation of mother and child possible | -0.28 | -0.08 | 0.35 | -0.87 | 0.31 |

$R^2 = 0.28$ ANOVA $*p < 0.05$

**Supplementary Table 3. Factors that affect the amount of change 〈POMS® 2 Depression-Dejection〉**

| Explanatory variable | Partial regression coefficient | Standard partial regression coefficient | P-value | 95%CI | |
|---|---|---|---|---|---|
| | | | | Min | Max |
| constant | -2.41 | | 0.05 | -4.75 | -0.06 |
| Intervention or not | -2.67 | -0.28 | 0.00* | -4.48 | -0.87 |
| Primipara | 0.63 | 0.07 | 0.54 | -1.38 | 2.64 |
| Delivery time: ≧8 hours | 0.15 | 0.02 | 0.89 | -1.94 | 2.25 |
| Blood loss during delivery: ≧500g | 0.04 | 0.00 | 0.97 | -1.87 | 1.95 |
| Separation of mother and child possible | 0.90 | 0.07 | 0.48 | -1.63 | 3.43 |

$R^2$ = 0.29 ANOVA *$p < 0.05$

**Supplementary Table 4. Factors that affect the amount of change 〈POMS® 2 Fatigue-Inertia〉**

| Explanatory variable | Partial regression coefficient | Standard partial regression coefficient | P-value | 95%CI | |
|---|---|---|---|---|---|
| | | | | Min | Max |
| constant | -3.61 | | 0.06 | -7.38 | 0.15 |
| Intervention or not | -3.58 | -0.23 | 0.02* | -6.48 | -0.69 |
| Primipara | -2.18 | -0.14 | 0.18 | -5.40 | 1.05 |
| Delivery time: ≧8 hours | -2.60 | -0.15 | 0.13 | -5.96 | 0.76 |
| Blood loss during delivery: ≧500g | 1.22 | 0.08 | 0.43 | -1.85 | 4.28 |
| Separation of mother and child possible | -2.72 | -0.12 | 0.19 | -6.78 | 1.35 |

$R^2$ = 0.12 ANOVA* $p < 0.05$

© 2026 Yamasaki A. et al.
